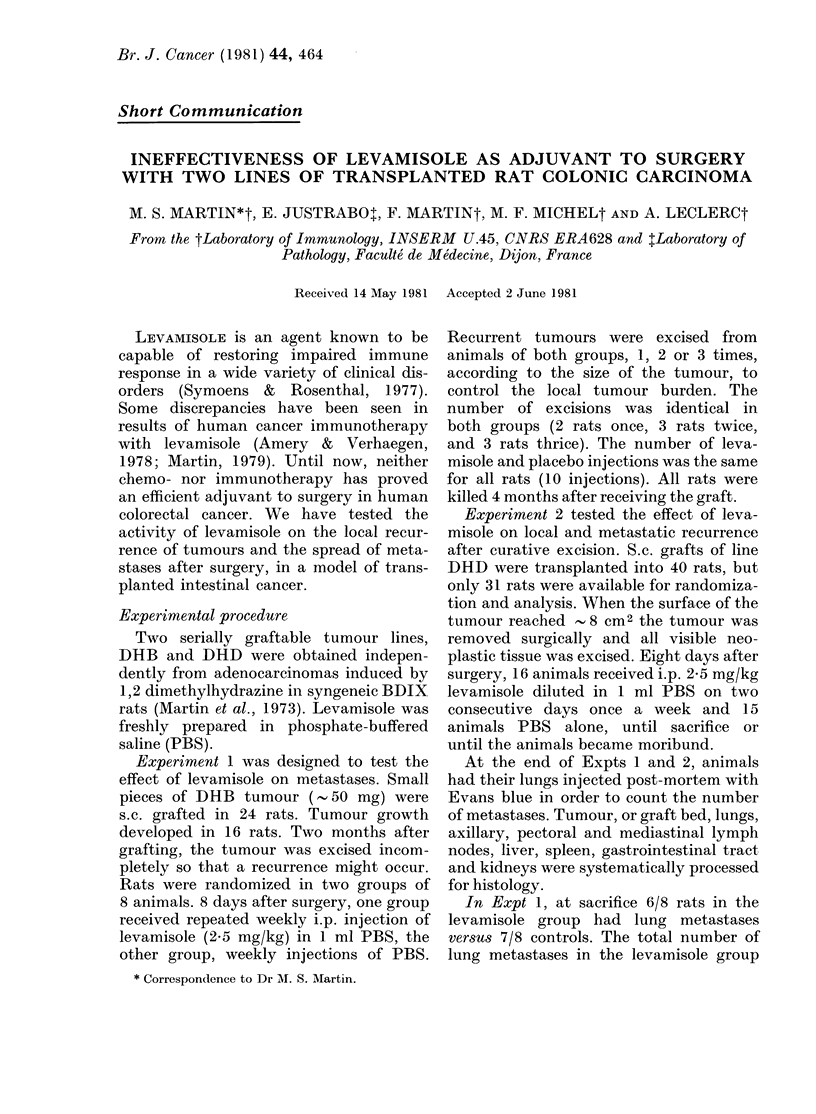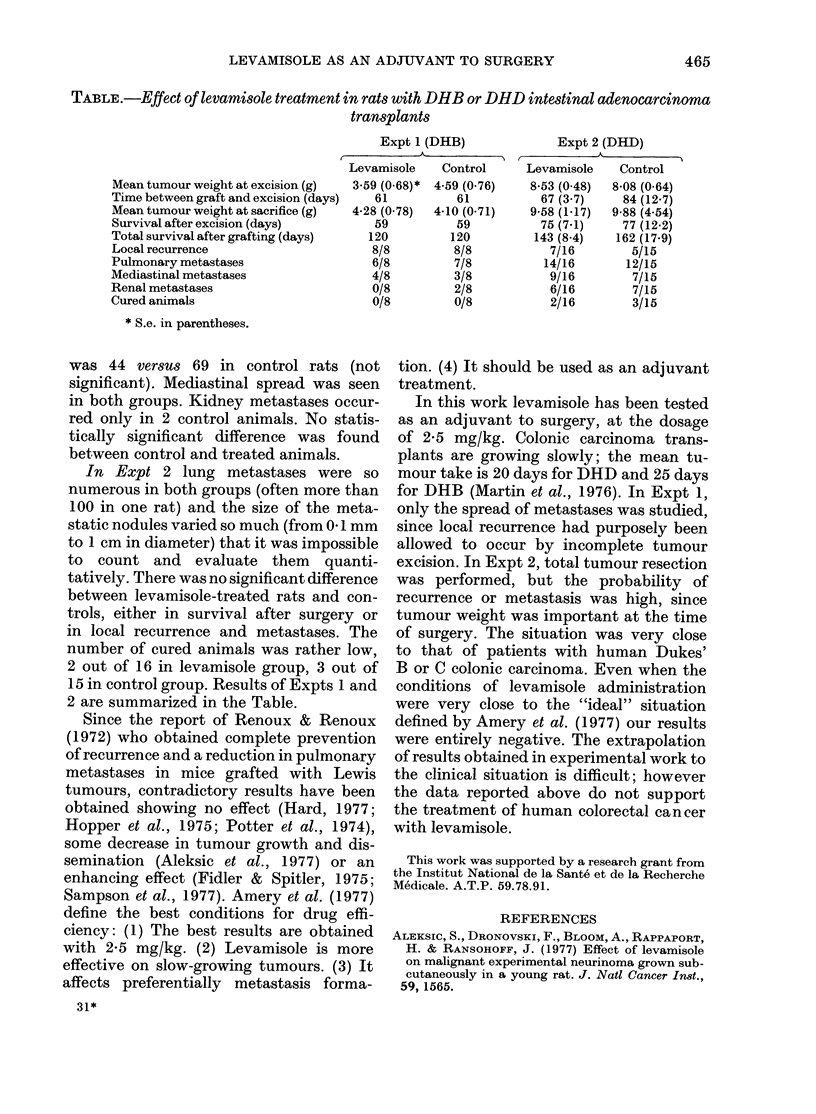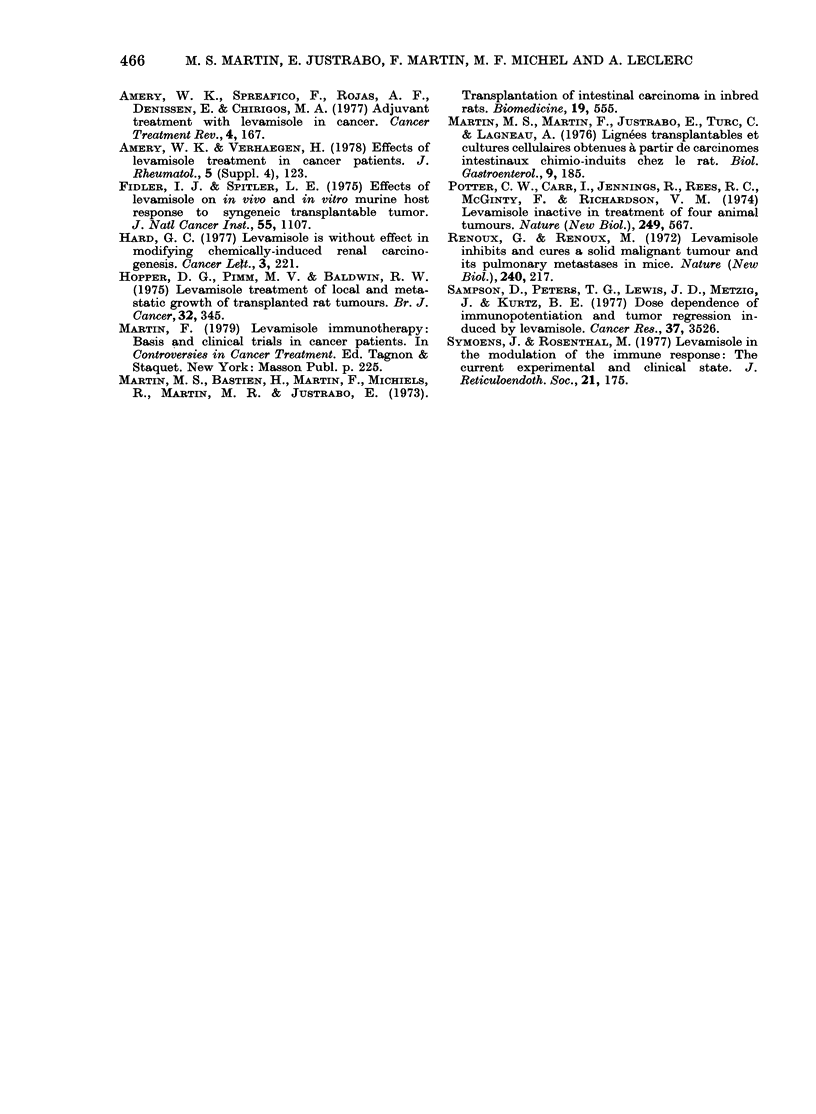# Ineffectiveness of levamisole as adjuvant to surgery with two lines of transplanted rat colonic carcinoma.

**DOI:** 10.1038/bjc.1981.207

**Published:** 1981-09

**Authors:** M. S. Martin, E. Justrabo, F. Martin, M. F. Michel, A. Leclerc


Br. J. Cancer (1981) 44, 464

*Short Communication*

### INEFFECTIVENESS OF LEVAMISOLE AS ADJUVANT TO SURGERY WITH TWO LINES OF TRANSPLANTED RAT COLONIC CARCINOMA

M. S. MARTIN*†, E. JUSTRABO‡, F. MARTIN†, M. F. MICHEL† AND A. LECLERC†

*From the †Laboratory of Immunology, INSERM U.45, CNRS ERA628 and ‡Laboratory of Pathology, Faculté de Médecine, Dijon, France*

Received 14 May 1981 Accepted 2 June 1981

LEVAMISOLE is an agent known to be capable of restoring impaired immune response in a wide variety of clinical disorders (Symoens & Rosenthal, 1977). Some discrepancies have been seen in results of human cancer immunotherapy with levamisole (Amery & Verhaegen, 1978; Martin, 1979). Until now, neither chemo- nor immunotherapy has proved an efficient adjuvant to surgery in human colorectal cancer. We have tested the activity of levamisole on the local recurrence of tumours and the spread of metastases after surgery, in a model of transplanted intestinal cancer.

*Experimental procedure*

Two serially graftable tumour lines, DHB and DHD were obtained independently from adenocarcinomas induced by 1,2 dimethylhydrazine in syngeneic BDIX rats (Martin *et al.*, 1973). Levamisole was freshly prepared in phosphate-buffered saline (PBS).

*Experiment* 1 was designed to test the effect of levamisole on metastases. Small pieces of DHB tumour ($\sim$50 mg) were s.c. grafted in 24 rats. Tumour growth developed in 16 rats. Two months after grafting, the tumour was excised incompletely so that a recurrence might occur. Rats were randomized in two groups of 8 animals. 8 days after surgery, one group received repeated weekly i.p. injection of levamisole (2·5 mg/kg) in 1 ml PBS, the other group, weekly injections of PBS. Recurrent tumours were excised from animals of both groups, 1, 2 or 3 times, according to the size of the tumour, to control the local tumour burden. The number of excisions was identical in both groups (2 rats once, 3 rats twice, and 3 rats thrice). The number of levamisole and placebo injections was the same for all rats (10 injections). All rats were killed 4 months after receiving the graft.

*Experiment* 2 tested the effect of levamisole on local and metastatic recurrence after curative excision. S.c. grafts of line DHD were transplanted into 40 rats, but only 31 rats were available for randomization and analysis. When the surface of the tumour reached $\sim$8 $cm^2$ the tumour was removed surgically and all visible neoplastic tissue was excised. Eight days after surgery, 16 animals received i.p. 2·5 mg/kg levamisole diluted in 1 ml PBS on two consecutive days once a week and 15 animals PBS alone, until sacrifice or until the animals became moribund.

At the end of Expts 1 and 2, animals had their lungs injected post-mortem with Evans blue in order to count the number of metastases. Tumour, or graft bed, lungs, axillary, pectoral and mediastinal lymph nodes, liver, spleen, gastrointestinal tract and kidneys were systematically processed for histology.

*In Expt* 1, at sacrifice 6/8 rats in the levamisole group had lung metastases *versus* 7/8 controls. The total number of lung metastases in the levamisole group

* Correspondence to Dr M. S. Martin.

TABLE.—*Effect of levamisole treatment in rats with DHB or DHD intestinal adenocarcinoma transplants*

| | Expt 1 (DHB) | | Expt 2 (DHD) | |
|---|---|---|---|---|
| | Levamisole | Control | Levamisole | Control |
| Mean tumour weight at excision (g) | 3·59 (0·68)* | 4·59 (0·76) | 8·53 (0·48) | 8·08 (0·64) |
| Time between graft and excision (days) | 61 | 61 | 67 (3·7) | 84 (12·7) |
| Mean tumour weight at sacrifice (g) | 4·28 (0·78) | 4·10 (0·71) | 9·58 (1·17) | 9·88 (4·54) |
| Survival after excision (days) | 59 | 59 | 75 (7·1) | 77 (12·2) |
| Total survival after grafting (days) | 120 | 120 | 143 (8·4) | 162 (17·9) |
| Local recurrence | 8/8 | 8/8 | 7/16 | 5/15 |
| Pulmonary metastases | 6/8 | 7/8 | 14/16 | 12/15 |
| Mediastinal metastases | 4/8 | 3/8 | 9/16 | 7/15 |
| Renal metastases | 0/8 | 2/8 | 6/16 | 7/15 |
| Cured animals | 0/8 | 0/8 | 2/16 | 3/15 |

* S.e. in parentheses.

was 44 *versus* 69 in control rats (not significant). Mediastinal spread was seen in both groups. Kidney metastases occurred only in 2 control animals. No statistically significant difference was found between control and treated animals.

*In Expt* 2 lung metastases were so numerous in both groups (often more than 100 in one rat) and the size of the metastatic nodules varied so much (from 0·1 mm to 1 cm in diameter) that it was impossible to count and evaluate them quantitatively. There was no significant difference between levamisole-treated rats and controls, either in survival after surgery or in local recurrence and metastases. The number of cured animals was rather low, 2 out of 16 in levamisole group, 3 out of 15 in control group. Results of Expts 1 and 2 are summarized in the Table.

Since the report of Renoux & Renoux (1972) who obtained complete prevention of recurrence and a reduction in pulmonary metastases in mice grafted with Lewis tumours, contradictory results have been obtained showing no effect (Hard, 1977; Hopper *et al.*, 1975; Potter *et al.*, 1974), some decrease in tumour growth and dissemination (Aleksic *et al.*, 1977) or an enhancing effect (Fidler & Spitler, 1975; Sampson *et al.*, 1977). Amery *et al.* (1977) define the best conditions for drug efficiency: (1) The best results are obtained with 2·5 mg/kg. (2) Levamisole is more effective on slow-growing tumours. (3) It affects preferentially metastasis formation. (4) It should be used as an adjuvant treatment.

In this work levamisole has been tested as an adjuvant to surgery, at the dosage of 2·5 mg/kg. Colonic carcinoma transplants are growing slowly; the mean tumour take is 20 days for DHD and 25 days for DHB (Martin *et al.*, 1976). In Expt 1, only the spread of metastases was studied, since local recurrence had purposely been allowed to occur by incomplete tumour excision. In Expt 2, total tumour resection was performed, but the probability of recurrence or metastasis was high, since tumour weight was important at the time of surgery. The situation was very close to that of patients with human Dukes' B or C colonic carcinoma. Even when the conditions of levamisole administration were very close to the "ideal" situation defined by Amery *et al.* (1977) our results were entirely negative. The extrapolation of results obtained in experimental work to the clinical situation is difficult; however the data reported above do not support the treatment of human colorectal cancer with levamisole.

This work was supported by a research grant from the Institut National de la Santé et de la Recherche Médicale. A.T.P. 59.78.91.

REFERENCES

ALEKSIC, S., DRONOVSKI, F., BLOOM, A., RAPPAPORT, H. & RANSOHOFF, J. (1977) Effect of levamisole on malignant experimental neurinoma grown subcutaneously in a young rat. *J. Natl Cancer Inst.*, **59**, 1565.

AMERY, W. K., SPREAFICO, F., ROJAS, A. F., DENISSEN, E. & CHIRIGOS, M. A. (1977) Adjuvant treatment with levamisole in cancer. *Cancer Treatment Rev.*, **4**, 167.

AMERY, W. K. & VERHAEGEN, H. (1978) Effects of levamisole treatment in cancer patients. *J. Rheumatol.*, **5** (Suppl. 4), 123.

FIDLER, I. J. & SPITLER, L. E. (1975) Effects of levamisole on *in vivo* and *in vitro* murine host response to syngeneic transplantable tumor. *J. Natl Cancer Inst.*, **55**, 1107.

HARD, G. C. (1977) Levamisole is without effect in modifying chemically-induced renal carcinogenesis. *Cancer Lett.*, **3**, 221.

HOPPER, D. G., PIMM, M. V. & BALDWIN, R. W. (1975) Levamisole treatment of local and metastatic growth of transplanted rat tumours. *Br. J. Cancer*, **32**, 345.

MARTIN, F. (1979) Levamisole immunotherapy: Basis and clinical trials in cancer patients. In *Controversies in Cancer Treatment*. Ed. Tagnon & Staquet. New York: Masson Publ. p. 225.

MARTIN, M. S., BASTIEN, H., MARTIN, F., MICHIELS, R., MARTIN, M. R. & JUSTRABO, E. (1973). Transplantation of intestinal carcinoma in inbred rats. *Biomedicine*, **19**, 555.

MARTIN, M. S., MARTIN, F., JUSTRABO, E., TURC, C. & LAGNEAU, A. (1976) Lignées transplantables et cultures cellulaires obtenues à partir de carcinomes intestinaux chimio-induits chez le rat. *Biol. Gastroenterol.*, **9**, 185.

POTTER, C. W., CARR, I., JENNINGS, R., REES, R. C., MCGINTY, F. & RICHARDSON, V. M. (1974) Levamisole inactive in treatment of four animal tumours. *Nature* (*New Biol.*), **249**, 567.

RENOUX, G. & RENOUX, M. (1972) Levamisole inhibits and cures a solid malignant tumour and its pulmonary metastases in mice. *Nature* (*New Biol.*), **240**, 217.

SAMPSON, D., PETERS, T. G., LEWIS, J. D., METZIG, J. & KURTZ, B. E. (1977) Dose dependence of immunopotentiation and tumor regression induced by levamisole. *Cancer Res.*, **37**, 3526.

SYMOENS, J. & ROSENTHAL, M. (1977) Levamisole in the modulation of the immune response: The current experimental and clinical state. *J. Reticuloendoth. Soc.*, **21**, 175.